# Preliminary Identification and Characterization of Antimicrobial Peptides from the Skin of *Rana amurensis* Based on Transcriptome Sequencing

**DOI:** 10.3390/biology15171479

**Published:** 2026-09-01

**Authors:** Tongtong Song, Xinning Zhang, Chen Wen, Fangyong Ning, Zhiheng Du, Qiushi Wang, Yuan Xu

**Affiliations:** 1 College of Animal Science and Technology, Northeast Agricultural University, No. 600 Changjiang Road, Xiangfang District, Harbin 150030, China; songtt010220@163.com (T.S.); zxn0502104@163.com (X.Z.); wen34820605@163.com (C.W.); ningfangyong@neau.edu.cn (F.N.); dzhh@neau.edu.cn (Z.D.); 2 Laboratory Animal Center, Affiliated Zhongshan Hospital of Dalian University, No. 6 Jiefang Street, Zhongshan District, Dalian 116001, China

**Keywords:** *Rana amurensis*, *Aeromonas hydrophila*, transcriptomics, toll-like receptor, antimicrobial peptides

## Abstract

The skin of *Rana amurensis* serves as an important defense barrier against microbial challenges, yet the molecular basis of its cutaneous immune defense and antimicrobial peptide (AMP) repertoire remains incompletely understood. Here, we established a skin transcriptomic profile of *R. amurensis* following *Aeromonas hydrophila* infection and integrated transcriptomic and bioinformatic approaches to characterize transcriptional responses and identify candidate AMPs. A total of 1669 differentially expressed genes (DEGs) were identified between the uninfected and infected groups, including 1091 up-regulated and 578 down-regulated genes. Infection with *A. hydrophila* induced marked transcriptional changes in innate immune-related genes and pathways, including differential expression of genes associated with the Toll-like receptor (TLR) signaling pathway. Transcriptome-based screening identified 151 candidate AMP sequences. Based on sequence characteristics, evolutionary conservation, and physicochemical properties (length ≤50 amino acids and net charge ranging from +2 to +9), six candidates were selected and three randomly selected candidates were chemically synthesized and exhibited antimicrobial activity against bacterial pathogens, inducing less than 10% hemolysis and maintaining L929 cell viability above 79% at 64 μg/mL. These findings indicate that *A. hydrophila* infection induces coordinated transcriptional responses involving innate immune-related pathways and diverse candidate AMPs in *R. amurensis*. This study supports the utility of transcriptome-guided discovery for identifying candidate AMPs in amphibians and provides insights into the molecular basis of skin defense in *R. amurensis*.

## Simple Summary

The skin of amphibians is an important barrier against harmful microbes, yet much remains to be learned about how it combats infection. In this study, we investigated how the skin of *Rana amurensis*, a frog native to northeastern Asia, responds when exposed to a common disease-causing bacterium. Transcriptome analysis revealed that the skin of these frogs contains an internal alarm system—a set of pathways involved in recognizing pathogens and mounting immune defenses. Furthermore, we used computational tools to search the transcriptome data for candidate antimicrobial peptides (AMPs) produced by the skin. In total, 151 candidate AMP sequences were identified; six of these were selected based on sequence characteristics, evolutionary conservation, and physicochemical properties, and three were synthesized and exhibited antimicrobial activity against bacterial pathogens. These findings improve our understanding of skin defense in *R. amurensis* and provide a resource for future studies of amphibian antimicrobial peptides.

## 1. Introduction

*Rana amurensis* (Amphibia: Anura: Ranidae) is a cold-adapted amphibian species widely distributed across the high-latitude regions of Northeast Asia, including northeastern China, Siberia, the Russian Far East, Mongolia, and North Korea [1,2]. This species occurs in diverse freshwater ecosystems, such as wetlands, ponds, marshes, and riparian environments, and is well adapted to seasonal temperature fluctuations and environmental challenges [3]. Long-term exposure to complex environmental conditions may have contributed to the evolution of specialized physiological and immune adaptations that maintain homeostasis and support pathogen defense.

Among infectious diseases affecting amphibians, red-leg syndrome, caused primarily by *Aeromonas hydrophila*, poses a major threat to frog populations due to its rapid transmission and high mortality [4,5,6]. The disease commonly affects both juvenile and adult frogs and is characterized by reduced activity, weakness, paralysis, and typical hemorrhagic lesions on the skin of the inner legs, thighs, and abdomen. In severe cases, infected frogs may develop extensive skin lesions, edema, and pathological changes in internal organs, including the liver, spleen, and kidneys [7,8]. The incidence of red-leg syndrome in forest frogs generally peaks between May and September [6]. A recent study showed that *A. hydrophila* infection induces changes in immune-related gene expression in multiple tissues of *R. amurensis*, including the skin [9]. Compared with the closely related species *Rana dybowskii*, *R. amurensis* is widely distributed across Heilongjiang Province and occurs as a dominant species in most surveyed areas [10], suggesting relatively strong environmental tolerance. As *R. amurensis* relies on aquatic habitats for breeding and hibernation [3,10], frequent exposure to aquatic microorganisms may have shaped its innate immune defenses. Nevertheless, the molecular basis of these responses to infection remains incompletely understood.

Amphibian skin secretions form an important chemical barrier against microbial invasion, with antimicrobial peptides (AMPs) serving as major effectors of innate immunity [11]. These bioactive peptides contribute to limiting microbial colonization and protecting amphibians from diverse environmental pathogens. Previous studies have identified several AMP families from the skin of *R. amurensis*. Zhou et al. isolated five AMP precursor genes from a skin cDNA library, including both known frog skin peptide homologs and structurally novel peptides [12]. Zhang et al. subsequently characterized Amurin-2 and its variants, showing antibacterial activity against *Staphylococcus aureus*, Candida albicans, and methicillin-resistant *S. aureus* (MRSA) [13]. Furthermore, Wang et al. identified 81 AMP-related cDNA sequences through degenerate PCR approaches, revealing considerable diversity within the AMP repertoire of *R. amurensis* [14]. These studies suggest that the skin AMPs of R. amurensis are an important component of its environmental adaptation and pathogen defense.

Despite these advances, most previously identified AMPs from *R. amurensis* were obtained using conventional molecular cloning and sequencing strategies, which cannot fully capture AMP diversity or pathogen-responsive expression patterns. Increasing evidence indicates that AMP production in amphibian skin is dynamically regulated by environmental conditions and microbial challenges. Therefore, a transcriptome-guided strategy following pathogen challenge may provide an effective approach for identifying additional AMP candidates and characterizing host immune responses. In this study, we established a skin transcriptomic profile of *R. amurensis* following *A. hydrophila* infection and performed integrated bioinformatic analyses to investigate transcriptional responses to infection and identify candidate AMPs. Our findings provide insights into amphibian skin immunity and expand the repertoire of candidate AMPs available for future studies.

## 2. Materials and Methods

### 2.1. Animals and Bacterial Strains

A total of 60 healthy adult male *Rana amurensis* were purchased from a local market in Harbin, Heilongjiang Province, China. The strain of *Aeromonas hydrophila* used in this study was provided by the Heilongjiang River Fisheries Research Institute, Chinese Academy of Fishery Sciences.

### 2.2. Preparation of Bacterial Suspension

*A. hydrophila* was recovered from a glycerol (Solarbio Science & Technology Co., Ltd., Beijing, China) stock, streaked onto LB agar plates, and incubated overnight. A single colony was then inoculated into 1 mL of LB broth and incubated at 28 °C with shaking at 180 rpm. Once the culture became turbid, the bacterial suspension was transferred into a larger volume of LB broth and incubated with shaking at 180 rpm and 28 °C for approximately 12 h. The culture was monitored at regular intervals until the OD600 reached 0.4–0.6, corresponding to approximately $1\times 10^{7}\;\mathrm{CFU}/\mathrm{mL}$. The bacterial cells were harvested by centrifugation at 4000 rpm for 10 min, washed twice with physiological saline, and resuspended in the same solution. The suspension was finally diluted to 10^6^ CFU/mL for use as the bacterial challenge inoculum.

### 2.3. A. hydrophila Challenge and Skin Secretion Collection

Six healthy adult *R. amurensis* individuals of similar body weight and size were randomly selected and divided into two groups: the control group (n = 3; ZA: A1, A3, A8) and the *A. hydrophila*-challenged group (n = 3; ZB: B1, B4, B8). For the *A. hydrophila*-challenged group, the frogs were immersed in 2500 mL of *A. hydrophila* suspension (10^6^ CFU/mL) for 36 h [15]. During this period, the method described by Li [16] was modified based on preliminary trials, and mild electrical stimulation (7 V, once every 10 min) was applied to the well-developed dorsal skin glands to induce skin secretion. The control group was immersed in physiological saline and subjected to the same electrical stimulation protocol. All frogs were then euthanized, and the entire skin was excised and stored at −80 °C until further analysis.

### 2.4. RNA Extraction and Quality Assessment

Total RNA was extracted from the skin tissues using TRIzol reagent (Invitrogen, Carlsbad, CA, USA). RNA integrity was initially evaluated by 1.2% agarose gel electrophoresis to assess RNA degradation and potential DNA contamination. RNA concentration was quantified using a Qubit 2.0 Fluorometer (Life Technologies, Carlsbad, CA, USA), and RNA integrity was further assessed with an Agilent 2100 Bioanalyzer (Agilent Technologies, Santa Clara, CA, USA) to ensure that all samples met the quality requirements for downstream sequencing.

### 2.5. cDNA Library Preparation and Illumina Sequencing

The cDNA library was constructed using an Illumina sequencing kit (Illumina, Inc., San Diego, CA, USA). Briefly, mRNA was enriched with Oligo(dT) magnetic beads and randomly fragmented in a divalent cation buffer. The fragmented mRNA was used as a template for first-strand cDNA synthesis using reverse transcriptase, followed by second-strand synthesis using DNA Polymerase I. After purification, end-repair, A-tailing, and adapter ligation, 370–420 bp cDNA fragments were selected with AMPure XP beads and amplified by PCR. Library quality was assessed using a Qubit 2.0 Fluorometer and Agilent 2100 Bioanalyzer. The libraries were quantified using qPCR (>2 nM) before pooling for 150 bp paired-end sequencing on the Illumina platform.

### 2.6. Sequence Data Processing and Analysis

Raw reads were processed using CASAVA (Illumina, Inc., San Diego, CA, USA) for base calling and filtered to remove adapter sequences, reads containing ambiguous bases (N), and low-quality reads (Phred score ≤ 20). High-quality clean reads were assembled with Trinity (v2.11.0) and validated via BUSCO. Transcripts were clustered into “Genes” with Corset (v4.6). Functional annotation was performed against the Nr, Nt, Pfam, Swiss-Prot, KOG, COG, KEGG, and GO databases. Differential expression analysis was performed using DESeq2 (v1.26.0), and functional enrichment was assessed with Goseq (v1.10.0) and KOBAS (v2.0.12). Candidate AMPs were identified via a pipeline incorporating TransDecoder (ORF extraction), SignalP (signal peptide prediction), and homology searches against CAMPR3 and DRAMP. Physicochemical properties and hemolytic potential were evaluated using ProtParam (https://web.expasy.org/protparam/; accessed on 3 November 2025) and HemoPI (http://crdd.osdd.net/raghava/hemopi/submitfreq.php?ran=5861; accessed on 7 November 2025), respectively. Three-dimensional structures were predicted using the AlphaFold2 web server [17] and visualized with Mol.* [18].

### 2.7. Prediction of Antimicrobial Peptides

We predicted AMP sequences using homology-based and pattern-matching approaches. First, transcripts containing a single open reading frame (ORF) were selected, and TransDecoder (v5.5.0) was used to identify the longest ORF. SignalP (v4.1) was used to predict signal peptides, and sequences longer than 150 amino acids were excluded. Next, hidden Markov models (HMMs) from CAMPR3 (https://camp3.bicnirrh.res.in/; accessed on 17 November 2025) [19] and homologous AMP sequences from the DRAMP database (https://dramp.cpu-bioinfor.org/static/help.php; accessed on 7 January 2026) [20] were used to predict AMPs. Sequences predicted to be non-antimicrobial by CS-AMPPred [21] using the default classification settings were excluded. In addition, following the strategy reported by Wang et al. (2020) [14], ten upstream primers were designed to target conserved regions of signal peptide sequences, and these primers were used to screen for AMP-encoding sequences within the assembled transcriptome.

### 2.8. Synthesis and Antimicrobial Activity Validation of Candidate AMPs

Selected candidate peptides were synthesized via solid-phase peptide synthesis (SPPS), purified by RP-HPLC, and confirmed by mass spectrometry (purity > 95%). Antimicrobial activity was evaluated using the agar disk diffusion method. *E. coli*, *Salmonella*, *S. aureus*, and *A. hydrophila* were cultured to 10^5^ CFU/mL and mixed with LB agar. Filter paper disks impregnated with synthesized peptide solutions at 1 mg/mL were placed onto the inoculated plates. The plates were incubated at 37 °C for 24 h, and antimicrobial activity was determined based on the presence or absence of a clear inhibition zone around each disk.

### 2.9. Minimum Inhibitory Concentration (MIC) Determination

The MIC values of the synthesized peptides were determined based on the microdilution method described in the Clinical and Laboratory Standards Institute (CLSI) guidelines [22], with appropriate modifications. Suspensions of *E. coli*, *Salmonella* sp., *A. hydrophila*, and *S. aureus* cultured for 18–24 h were resuspended in fresh LB medium to approximately 1 × 10^6^ CFU/mL. First, 100 μL of the peptide stock solution (1024 μg/mL) was added to column 1 of a sterile 96-well microtiter plate, and 50 μL of ultrapure water was added to columns 2–10. Two-fold serial dilutions were performed by transferring 50 μL from column 1 to column 2, mixing, and continuing through column 8. After discarding 50 μL from column 8, the peptide concentrations before bacterial inoculation were 1024, 512, 256, 128, 64, 32, 16, and 8 μg/mL. Subsequently, 50 μL of the bacterial suspension was added to each well of columns 1–9. Column 9, containing 50 μL of ultrapure water and 50 μL of bacterial suspension, served as the growth control, whereas column 10, containing 50 μL of ultrapure water and 50 μL of LB medium, served as the blank control. Because an equal volume of the bacterial suspension was added to the peptide dilution series, the final peptide concentrations in columns 1–8 were 512, 256, 128, 64, 32, 16, 8, and 4 μg/mL, respectively, and the final bacterial density was approximately 5 × 10^5^ CFU/mL. The plate was incubated at 37 °C for 24 h, and bacterial growth was then assessed visually. The assay was considered valid when normal bacterial growth was observed in the growth control and the blank control remained clear. The MIC was defined as the lowest final peptide concentration that completely inhibited visible bacterial growth. Each assay was independently repeated three times.

### 2.10. Hemolysis Assay

The hemolytic activity of the peptides was evaluated using mouse erythrocytes with minor modifications to a previously described method [23]. Blood was collected from the mouse tail vein and immediately mixed with an equal volume of Alsever’s solution. The mixture was centrifuged at 1000× *g* for 5 min and the supernatant was discarded. The erythrocyte pellet was washed repeatedly with sterile physiological saline until the supernatant became clear, and the cells were then resuspended in physiological saline at a density of 1 × 10^7^–10^8^ cells/mL. Aliquots of 90 μL of the erythrocyte suspension were mixed with 10 μL of peptide solution at concentrations of 16–512 μg/mL and incubated at 37 °C for 30 min. After centrifugation at 1000× *g* for 5 min, the absorbance of the supernatant was measured at 540 nm using a microplate reader (BioTek Instruments, Inc., Winooski, VT, USA). Physiological saline served as the negative control (0% hemolysis) and 1% Triton X-100 as the positive control (100% hemolysis). Each treatment was performed in triplicate. The hemolysis rate was calculated as follows:

Hemolysis (%) = (OD_540_ sample − OD_540_ negative control)/(OD_540_ positive  control − OD_540_ negative control) × 100

### 2.11. Cytotoxicity Assay

The cytotoxicity of the peptides toward mammalian cells was evaluated using the mouse fibroblast cell line L929 (Procell Life Science & Technology Co., Ltd., Wuhan, China) and a Cell Counting Kit-8 (CCK-8; Beyotime Biotechnology, Shanghai, China) assay with minor modifications to a previously described method [24]. L929 cells were collected by trypsinization (Solarbio Science & Technology Co., Ltd., Beijing, China), centrifuged at 1000 rpm for 5 min, and resuspended in complete DMEM (Solarbio Science & Technology Co., Ltd., Beijing, China). The cell suspension was counted and adjusted so that each well of a 96-well plate received 100 μL containing approximately 5000 cells. The perimeter wells of the plate were filled with sterile water to minimize edge effects. After incubation at 37 °C in a humidified atmosphere containing 5% CO_2_ for 24 h, the serum-containing medium was removed and replaced with 100 μL of peptide solution per well. Peptide solutions were first prepared at 512 μg/mL in DMEM and then serially diluted two-fold to obtain five concentrations (512–32 μg/mL). After a further 24 h of incubation, 10 μL of CCK-8 solution was added to each well in the dark, and the plates were incubated at 37 °C for 1.5 h. The absorbance was then measured at 450 nm using a microplate reader. Wells containing culture medium without cells served as blank controls, and wells containing untreated cells served as negative controls. Each treatment was performed in triplicate. Cell viability was calculated as follows:

Cell viability (%) = (OD_450_ sample − OD_450_ blank)/(OD_450_ negative control −  OD_450_ blank) × 100

### 2.12. Data Analysis

Data from the hemolysis and cytotoxicity assays were analyzed using GraphPad Prism (version 10.1.2; GraphPad Software, San Diego, CA, USA) and are expressed as mean ± standard deviation (SD) (n = 3). Differences were considered statistically significant at * *p* < 0.05, ** *p* < 0.01; ns, not significant.

## 3. Results

### 3.1. Sample Collection and RNA Quality Assessment

During the challenge, no deaths or visible signs of skin infection were observed. Three frogs were therefore sampled from each of the control and challenged groups, and skin tissues were collected for RNA extraction. Total RNA was extracted from the skin tissues of the control group and the *A. hydrophila*-challenged group. The quality of the extracted RNA was assessed using an Agilent 2100 Bioanalyzer, and all samples were suitable for downstream sequencing.

### 3.2. Transcriptome Sequencing and Assembly

**Figure 1:**
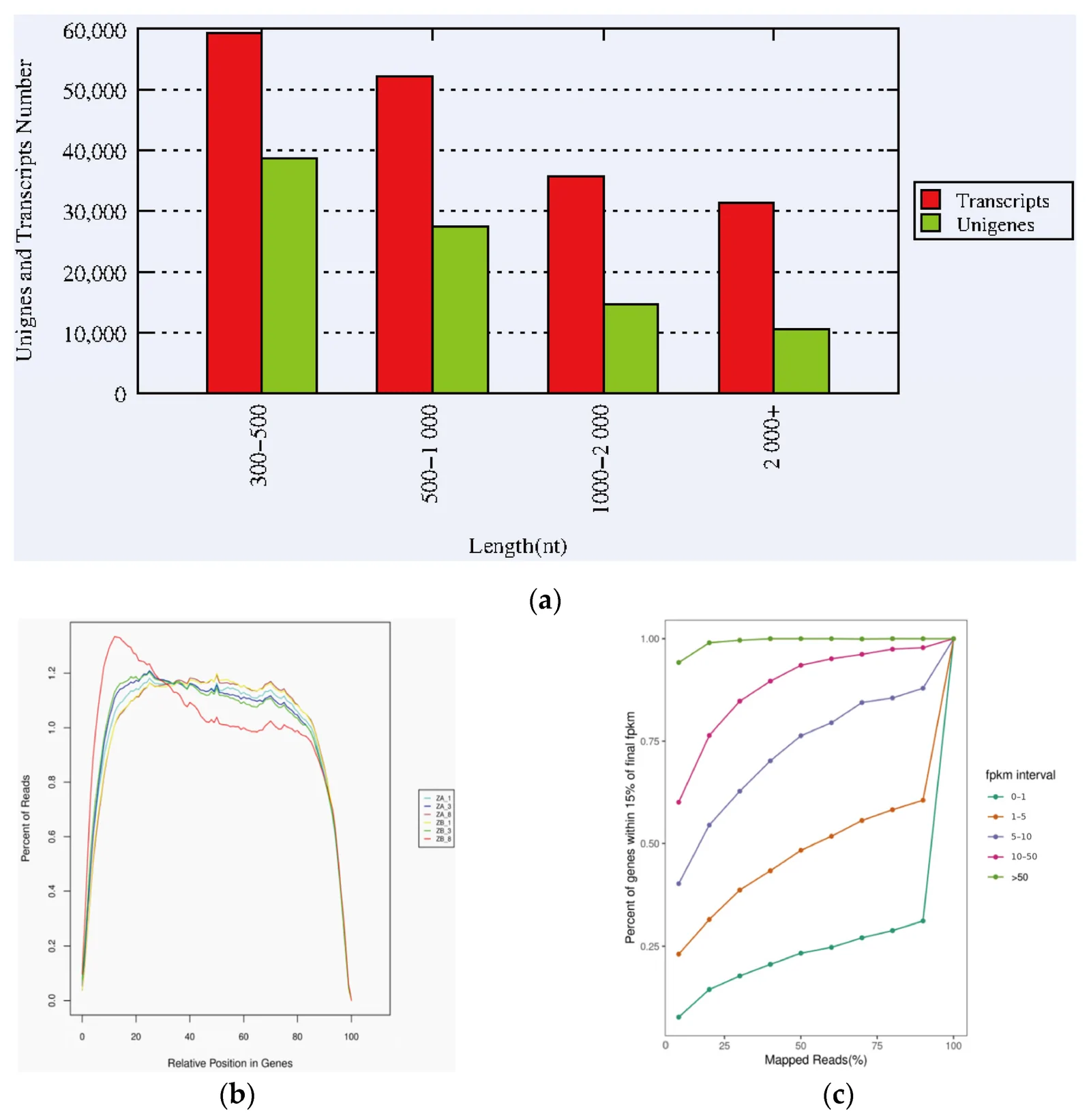
Quality assessment of *de novo* transcriptome assembly and sequencing data. (**a**) Length distribution of transcripts and unigenes; (**b**) Distribution of mapped reads across normalized gene positions, showing the randomness of mRNA fragmentation; (**c**) Saturation analysis of transcriptome sequencing data, demonstrating the percentage of detected genes within 15% of final FPKM values relative to the sequencing depth.

Sequencing on the Illumina HiSeq 4000 platform yielded a total of 136.97 million high-quality clean reads (Table 1), with a base error rate below 1%. The Q20 and Q30 values exceeded 97% and 92%, respectively, indicating high base-calling accuracy. De novo assembly yielded 178,381 transcripts and 91,313 unigenes, with N50 values of 2065 bp and 1517 bp, respectively (Figure 1a), reflecting high assembly integrity. To assess library construction bias and sequencing depth, the randomness of mRNA fragmentation and data saturation were evaluated. The distribution of mapped reads across mRNA transcripts (Figure 1b) showed a random pattern, indicating no significant bias during library construction. The saturation curves (Figure 1c) reached a plateau, with the majority of genes exhibiting FPKM values ≥ 0.1, suggesting that the sequencing depth was sufficient to capture transcriptome complexity and support accurate downstream gene quantification.

### 3.3. Functional Annotation and Classification

A total of 255,751 transcripts were searched against the KOG, KEGG, Nr, GO, and Swiss-Prot databases. Among these, 41,664 were annotated against the Nr database, and 16,987 were matched to Swiss-Prot (Table 2).

### 3.4. Gene Enrichment Analysis

**Figure 2:**
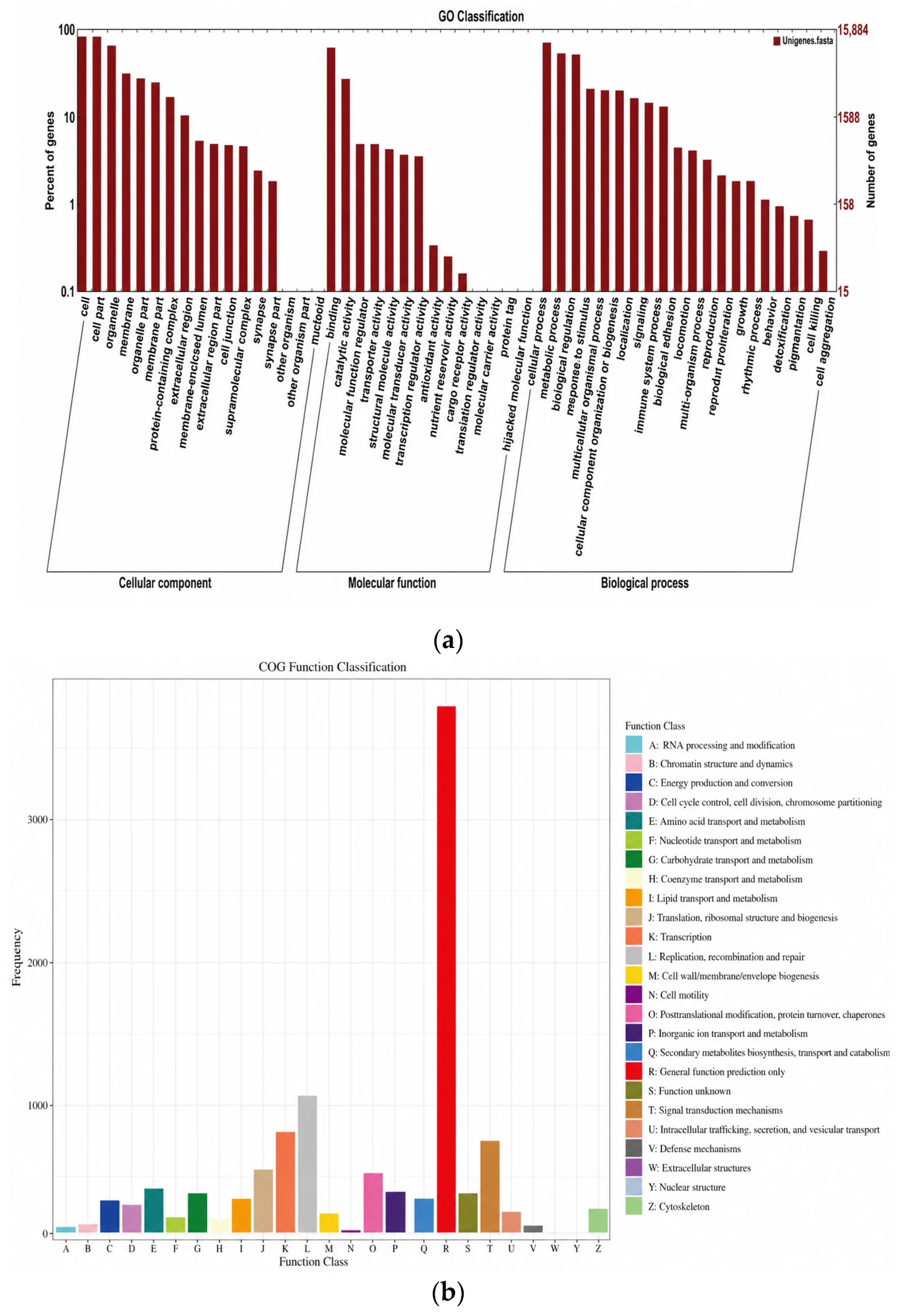

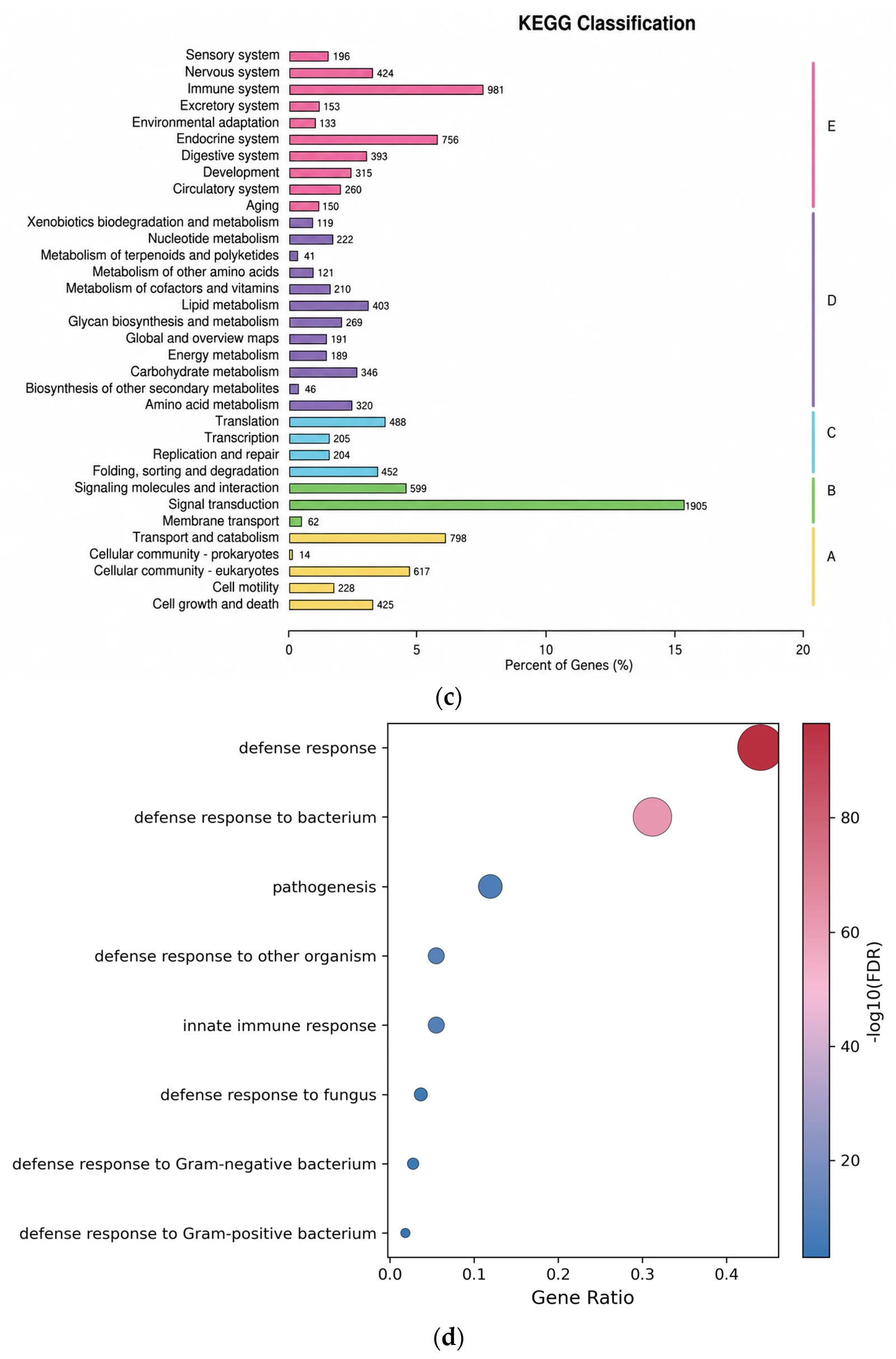
Functional annotation and classification of assembled transcripts. (**a**) GO annotation classification of transcripts. (**b**) COG functional classification of transcripts. (**c**) KEGG pathway classification of transcripts. A–E denote cellular processes, environmental information processing, genetic information processing, metabolism, and organismal systems, respectively. (**d**) GO enrichment analysis of AMP-associated genes. Bubble size indicates the number of AMP-associated genes in each enriched term, and color intensity represents −log10(FDR).

Gene Ontology (GO) annotation was obtained for 15,884 unigenes, with 84,731, 64,084, and 26,131 terms assigned to the biological process, cellular component, and molecular function categories, respectively (Figure 2a). COG (Clusters of Orthologous Groups) analysis classified the gene products into orthologous groups, with the majority of unigenes assigned to the “General function prediction only” category (Figure 2b). KEGG (Kyoto Encyclopedia of Genes and Genomes) pathway analysis mapped 12,235 unigenes to 34 pathways across five functional branches: organismal systems, metabolism, genetic information processing, environmental information processing, and cellular processes (Figure 2c). The immune system category contained 2320 unigenes, ranking second only to signal transduction (Figure 2c). GO enrichment analysis of the 110 AMP-related genes showed significant enrichment (FDR < 0.05) in eight biological process terms: “defense response”, “defense response to bacterium”, “pathogenesis”, “defense response to other organism”, “innate immune response”, “defense response to fungus”, “defense response to Gram-negative bacterium”, and “defense response to Gram-positive bacterium” (Figure 2d).

### 3.5. Prediction of Coding Sequences and Differential Expression Analysis

**Figure 3:**
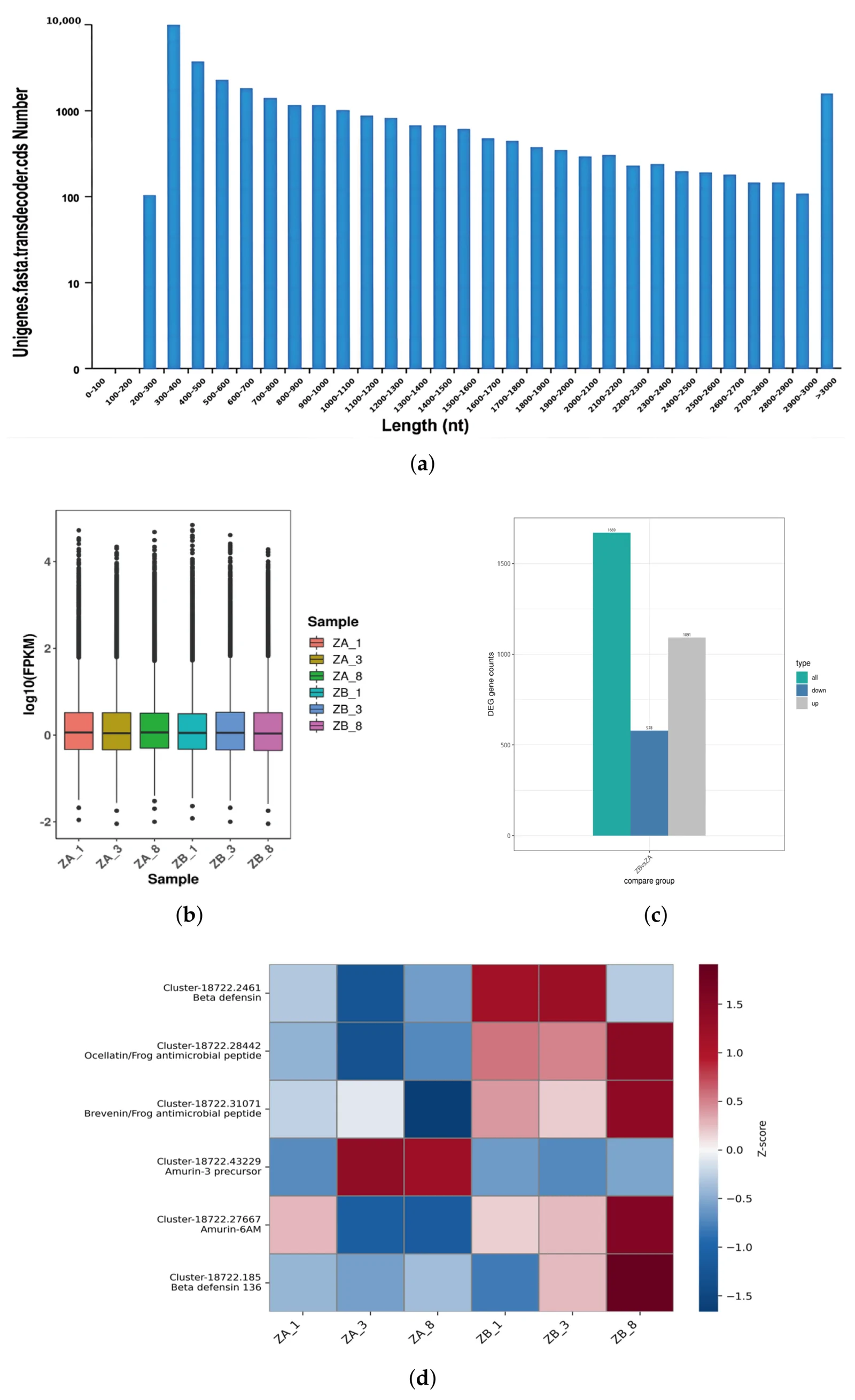
CDS length distribution, gene expression profiling, and differential expression analysis. (**a**) Length distribution of predicted CDSs. (**b**) Box plot of log10(FPKM) values across samples. (**c**) Number of DEGs between the ZA and ZB groups. (**d**) Heatmap showing expression profiles of the six AMP-related DEGs.

TransDecoder (v5.5.0) was used to identify potential coding sequences (CDSs) based on ORF length, log-likelihood scores, and sequence alignments against the Pfam database. The CDS length distribution peaked at 300–400 bp (Figure 3a). For gene expression analysis, clean reads were mapped to the unigene library using Bowtie2 (v2.4.4), and expression levels were estimated as FPKM values using RSEM. The box plot of expression distributions (Figure 3b) showed similar expression levels among samples, indicating consistent sequencing and expression profiles. Differential expression analysis identified 1669 differentially expressed genes (DEGs) between the ZA and ZB groups (Figure 3c). Compared with the ZA group, 1091 genes were upregulated and 578 genes were downregulated in the ZB group. Several immune-related DEGs were identified, including transcripts encoding cytokines, pattern-recognition receptors, complement components, and AMPs (Table S1). Interleukin-32 was significantly upregulated (log2FC = 1.90, q = 0.027), whereas *TLR13* and complement component C7 showed trends toward upregulation without reaching statistical significance. Three DEGs were annotated to the Toll-like receptor signaling pathway: *MAP2K7*, *JUN*, and *TLR13* (Table S2). Six of the AMP-related genes were identified among the DEGs (Table 3): Beta defensin, Ocellatin/Frog antimicrobial peptide, Brevenin/Frog antimicrobial peptide, Amurin-3 precursor, Amurin-6AM, and Beta defensin 136. Beta defensin (log2FC = 2.69, q = 0.065) and Ocellatin (log2FC = 1.43, q = 0.056) were upregulated, whereas Amurin-3 precursor was downregulated (log2FC = −6.74, q = 0.052). The expression profiles of these six AMP transcripts are shown in a heatmap (Figure 3d).

### 3.6. Identification and Screening of Antimicrobial Peptides

An integrated screening strategy combining Hidden Markov Model (HMM) searching, homology alignment, and pattern-based matching was used to identify candidate AMPs from the transcriptome data. First, 310,457 assembled transcripts were screened to identify 36,861 candidate coding sequences (CDSs) containing N-terminal signal peptides using TransDecoder. Potential AMPs were then identified through three complementary approaches: HMM searching against CAMPR3 (45 sequences), homology alignment against the DRAMP database (67 sequences), and pattern-based matching with known AMP sequences (68 sequences). After merging and removing redundant sequences, 171 unique candidates were obtained. Subsequent evaluation using the CS-AMPPred machine learning tool yielded 79 high-confidence AMP candidates (Table 4). Independently, following the signal peptide conserved-region strategy reported by Wang et al. [14], we identified an additional 72 candidate AMPs by primer-based screening of the assembled transcriptome. In total, 151 candidate AMPs (79 + 72) were identified from the skin of *R. amurensis* (Table 5).

### 3.7. Phylogenetic Analysis and Physicochemical Characterization of Antimicrobial Peptides

**Figure 4:**
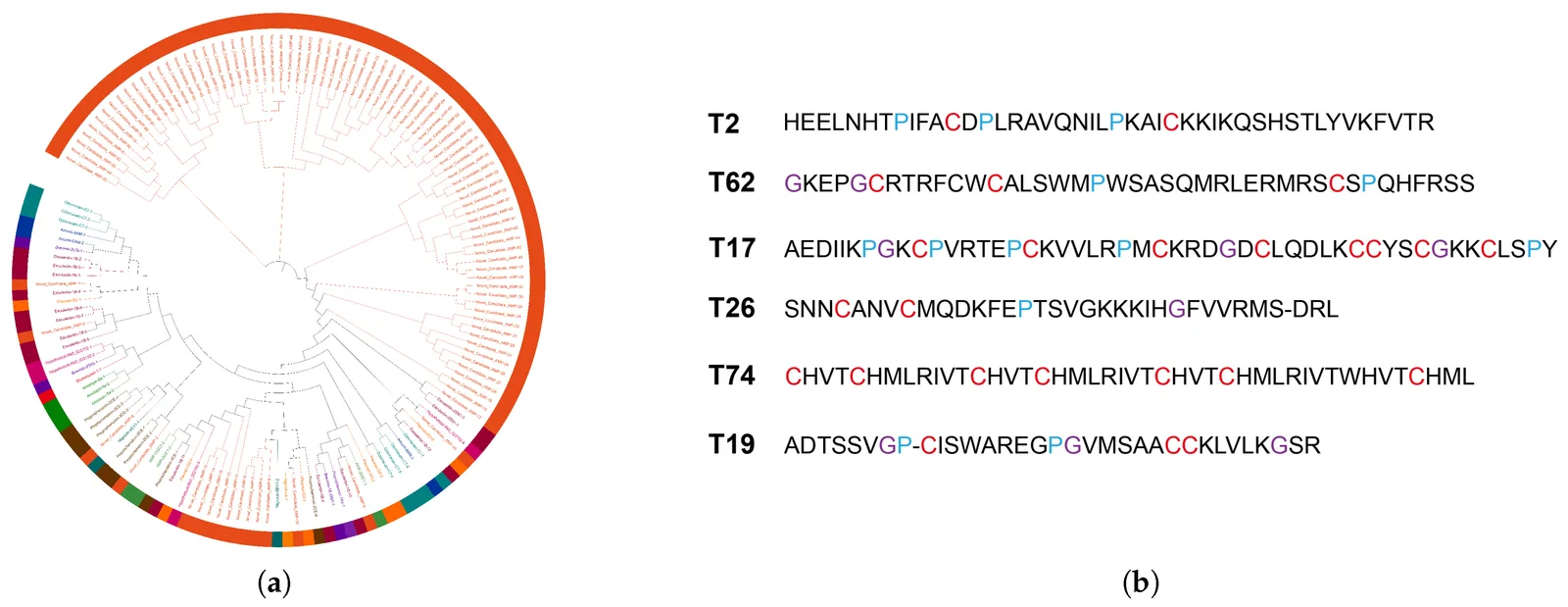
Phylogenetic analysis and sequence composition of *Rana amurensis* skin antimicrobial peptides. (**a**) Phylogenetic tree of the identified AMP cDNA sequences. Different colors indicate different AMP families, whereas the novel candidate AMPs are shown in orange. (**b**) Amino acid composition of mature AMPs (length ≤ 50 amino acids), highlighting the distribution of conserved residues.

Phylogenetic analysis of 151 predicted AMP sequences from *R. amurensis* showed multiple distinct clusters corresponding to known amphibian AMP families and previously uncharacterized groups (Figure 4a). A linear representation of the phylogenetic tree is shown in Figure S1. Among these sequences, 55 candidates (36.4%) clustered with well-characterized AMP families, including Amurin-6AM, Esculentin-1B, and Preprochensinin-2CE, indicating close evolutionary relationships with previously reported amphibian AMPs. The remaining 96 sequences (63.6%) formed separate clusters without clear assignment to well-characterized AMP families, suggesting that they may represent new AMP candidates. Combined bioinformatic and primer-based screening identified 90 non-redundant mature AMP sequences (Table 6). Further physicochemical screening identified six candidate mature peptides that met the criteria of length ≤50 amino acids and net charge ranging from +2 to +9, designated T2, T17, T19, T26, T62, and T74. These candidate peptides were rich in proline (P), cysteine (C), and glycine (G), with molecular weights ranging from 3,337.89 to 5,492.60 Da and isoelectric points (pI) between 8.63 and 9.88, consistent with their cationic properties (Table 7, Figure 4b). Stability analysis showed that T62 had a predicted in vivo half-life of >10 h, while T74 and T2 were predicted to be thermally stable. The GRAVY index (−0.29 to 0.00) and amphiphilicity index (maximum value, 1.11) indicated that these peptides possess a balance of hydrophobic and hydrophilic properties. Although PROB scores suggested a potential hemolytic risk, the predicted physicochemical properties of these peptides provide a basis for further investigation (Table 8).

### 3.8. Antimicrobial Activity of the Synthesized Peptides

Three of the six candidate peptides (T2, T19, and T26) were randomly selected for chemical synthesis to assess their antimicrobial activity. The synthetic peptides were purified by reversed-phase high-performance liquid chromatography (RP-HPLC) and verified by electrospray ionization mass spectrometry (ESI-MS). The predicted three-dimensional structures of the three synthesized peptides are shown in Figure S2. The antimicrobial activity of the three peptides was first assessed using the disk diffusion assay against four bacterial strains: *Escherichia coli*, *Staphylococcus aureus*, *Salmonella* sp., and *Aeromonas hydrophila* (Figure S3). All three peptides produced inhibition zones against the Gram-negative strains tested (*E. coli*, *Salmonella* sp., and *A. hydrophila*). In contrast, only T19 showed an inhibition zone against the Gram-positive bacterium *S. aureus* (Table 9). The disk diffusion assay provided only a qualitative assessment of antimicrobial activity. The MICs of the three peptides were therefore determined against the four strains (Table 10, Table 11 and Table 12). T19 and T26 inhibited *E. coli* with MICs of 64 μg/mL, lower than that of T2 (512 μg/mL). For *Salmonella* sp., T26 showed an MIC of 64 μg/mL, lower than those of T2 and T19 (both 128 μg/mL). For *A. hydrophila*, the MIC of T2 was 64 μg/mL, lower than those of T19 and T26 (both 128 μg/mL). Against *S. aureus*, only T19 exhibited inhibitory activity within the tested concentration range, with an MIC of 64 μg/mL, whereas T2 and T26 showed no detectable inhibition at the highest concentration tested (512 μg/mL). Overall, the three peptides displayed different inhibitory activities against the tested strains.

### 3.9. Hemolytic and Cytotoxic Activities of the Synthesized Peptides

**Figure 5:**
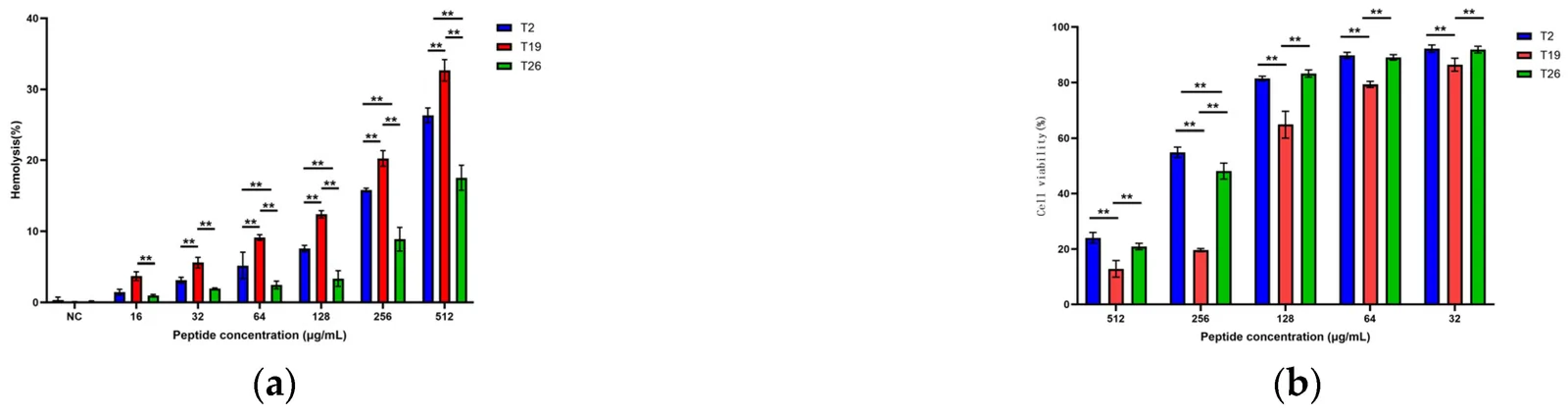
The hemolytic activity of T2, T19, and T26 on mouse erythrocytes (**a**) and their effects on L929 cell viability (**b**). Statistical significance was analyzed using two-way ANOVA followed by Tukey’s multiple comparisons test. ** *p* < 0.01.

The hemolytic and cytotoxic activities of T2, T19, and T26 were evaluated using mouse erythrocytes and the mouse fibroblast cell line L929, respectively. All three peptides exhibited concentration-dependent hemolytic activity over the tested concentration range (16–512 μg/mL) (Figure 5a). Hemolysis increased gradually with increasing peptide concentration, reaching 26.33 ± 1.04%, 32.65 ± 1.51%, and 17.54 ± 1.75% for T2, T19, and T26, respectively, at 512 μg/mL. At concentrations up to 128 μg/mL, hemolysis remained below 13% for all three peptides. Two-way ANOVA followed by Tukey’s multiple comparisons test showed that T19 induced significantly higher hemolysis than T2 and T26 at each tested concentration (*p* < 0.01). T26 exhibited the lowest hemolytic activity among the three peptides: its hemolysis was significantly lower than that of T19 at all tested concentrations and lower than that of T2 at 64 μg/mL and above (*p* < 0.01) (Figure 5a). In the CCK-8 assay, all three peptides caused a concentration-dependent reduction in L929 cell viability over the tested concentration range (32–512 μg/mL) (Figure 5b). At the highest concentration tested (512 μg/mL), cell viability decreased to 24.07 ± 1.95%, 12.85 ± 2.97%, and 20.91 ± 1.14% following treatment with T2, T19, and T26, respectively. At the lowest concentration tested (32 μg/mL), cell viability remained above 80% for all three peptides. Two-way ANOVA followed by Tukey’s multiple comparisons test showed that T19 induced a significantly greater reduction in L929 cell viability than T2 and T26 at each tested concentration (*p* < 0.01), whereas T2 and T26 did not differ significantly except at 256 μg/mL (*p* < 0.01) (Figure 5b). Overall, T26 exhibited the lowest hemolytic activity, whereas T19 showed the highest hemolytic and cytotoxic activities among the three peptides.

## 4. Discussion

The skin of amphibians is a multifunctional barrier that provides physical protection and contributes to immune regulation under environmental challenges [24,25,26]. Owing to their permeable skin and frequent contact with aquatic microorganisms, amphibians are continuously exposed to diverse potential pathogens and therefore rely on innate immune defenses [27,28]. Beyond serving as a physical barrier, amphibian skin contains specialized dermal granular glands that secrete a variety of bioactive molecules, including AMPs, immune-related peptides, and wound-healing factors, which collectively contribute to host defense and physiological homeostasis [26,28]. Previous transcriptomic studies have shown that amphibian skin contains abundant transcripts encoding bioactive peptides, including defensins, wound-healing peptides, and thymosin-related peptides, many of which have conserved biological functions across amphibian taxa [29,30]. In the present study, transcriptomic analysis of *Rana amurensis* skin following *Aeromonas hydrophila* challenge showed significant enrichment of immune-related biological processes. The enrichment of “immune response”, ‘immune system process’, and ‘innate immune response’ terms suggests that the skin of *R. amurensis* is involved in pathogen-associated transcriptional responses rather than functioning solely as a passive barrier. These findings indicate that skin immunity plays an important role in amphibian health and host defense.

The innate immune system serves as the first line of defense against invading microorganisms, and Toll-like receptor (TLR) signaling contributes to pathogen recognition and the initiation of immune responses [31,32,33]. TLRs recognize conserved microbe-associated molecular patterns (MAMPs) and initiate signaling cascades involved in inflammatory responses and antimicrobial defense. *Aeromonas hydrophila* is a major opportunistic pathogen of amphibians and is commonly associated with red-leg syndrome, a disease characterized by severe tissue damage and high mortality [4,5,6]. The pathogenicity of *A. hydrophila* is associated with multiple virulence factors, including lipopolysaccharides (LPS), exotoxins, and adhesion factors [34]. Previous studies have shown that bacterial challenge affects the expression of multiple TLR family members in amphibians, including *TLR2*, *TLR4*, *TLR5*, and *TLR9* [33,35]. In the present study, several DEGs associated with the TLR signaling pathway, including *MAP2K7*, *JUN*, and *TLR13*, were identified in the skin of *R. amurensis* following *A. hydrophila* challenge, suggesting a potential involvement of TLR-associated responses in antibacterial defense. Given that *R. amurensis* inhabits freshwater environments with frequent microbial exposure, changes in innate immune-related genes and antimicrobial peptide transcripts may contribute to its responses to bacterial challenge. Together, these findings provide insights into skin defense responses following bacterial challenge in *R. amurensis*.

Skin-derived antimicrobial peptides (AMPs) are important chemical defense components in amphibians and help limit microbial colonization and infection. The diversity of amphibian AMPs is shaped by both evolutionary history and environmental pressures. Earlier studies have shown that closely related frog species may possess distinct AMP repertoires, potentially reflecting adaptation to different ecological conditions. For example, Cortázar-Chinarro et al. characterized the skin AMPs of *Rana arvalis* and *Rana temporaria* populations and observed marked variation in AMP sequences across geographic regions, suggesting that AMP diversity may support adaptation to local environments [36]. Similarly, Wang et al. analyzed the skin transcriptomes of three sympatric frog species in Northeast China and identified substantial differences in AMP family composition, with 14 AMP families detected in *R. amurensis*, 7 in *Rana dybowskii*, and 13 in *Pelophylax nigromaculatus* [14]. These findings suggest that species-specific AMP repertoires may contribute to amphibian adaptation to diverse microbial environments.

Although previous studies have identified several AMP families in *R. amurensis* using molecular cloning and peptide sequencing, these conventional methods may capture only a subset of expressed antimicrobial molecules and therefore provide limited coverage of AMP diversity under pathogen challenge [12,13,14]. In contrast, transcriptomic approaches enable the expression of host defense molecules to be characterized under defined physiological or environmental conditions [37,38,39]. In this study, transcriptome-based screening of *R. amurensis* skin following *A. hydrophila* challenge identified 151 candidate AMP sequences, expanding the pool of putative AMP sequences for further investigation in this species. These candidates exhibited considerable sequence diversity and spanned multiple AMP-related lineages in *R. amurensis*.

Computational prediction alone cannot establish the biological activity of candidate AMPs. Therefore, functional validation is essential for assessing their antimicrobial potential [13,14]. In this study, six candidates were selected based on sequence characteristics, evolutionary conservation, and physicochemical properties. Three candidates were randomly selected for chemical synthesis, and all exhibited antimicrobial activity against bacterial pathogens. At 64 μg/mL, all three peptides induced less than 10% hemolysis and maintained L929 cell viability above 79%, whereas their MICs ranged from 64 to 512 μg/mL. These findings support the utility of transcriptome-guided discovery combined with bioinformatic screening for identifying candidate antimicrobial peptides in amphibians [37,39]. Compared with conventional AMP discovery approaches, this integrated strategy enables the simultaneous investigation of pathogen-associated transcriptional responses and exploration of candidate antimicrobial peptides, providing a useful framework for studying skin defense in non-model amphibians.

Taken together, these findings indicate that *A. hydrophila* infection induces coordinated transcriptional responses involving innate immune-related pathways and diverse candidate AMPs in *R. amurensis*. Differential expression of genes associated with the TLR signaling pathway, together with the identification of diverse AMP candidates, provides new insights into the molecular basis of skin defense in *R. amurensis*. Given the increasing threats posed by infectious diseases and environmental change to amphibian populations worldwide [4,6], understanding natural immune defenses is important for advancing our knowledge of amphibian health and host defense. Furthermore, the AMP candidates identified in this study provide a useful resource for future studies of natural antimicrobial molecules and host–microbe interactions in amphibians.

This study represents a preliminary exploration of the skin antimicrobial peptides of *R. amurensis* under A. hydrophila challenge based on transcriptome sequencing. The analysis identified 151 AMP candidate sequences, and the three randomly selected for chemical synthesis all exhibited antimicrobial activity against the tested bacterial pathogens. Despite these findings, several aspects warrant further investigation. First, only three of the six identified candidates were chemically synthesized, and the remaining three have not been tested. Second, the synthesized peptides were not compared with well-characterized AMPs under identical conditions and were assessed only in vitro. Future studies should include established AMPs as positive controls, evaluate their in vivo efficacy and safety, and clarify their antibacterial mode of action through membrane permeabilization assays and electron microscopy. Third, skin samples were collected at a single time point (36 h) after challenge, so temporal changes in AMP expression were not captured. Transcriptomic analyses at multiple post-challenge time points are needed to delineate these changes.

## 5. Conclusions

This study characterized the skin immune response of *Rana amurensis* to *Aeromonas hydrophila* challenge using transcriptome profiling. The challenge induced marked transcriptional changes in innate immune-related genes and pathways, including genes associated with the Toll-like receptor (TLR) signaling pathway. Transcriptome-based screening identified 151 candidate AMP sequences. Six candidates were selected based on sequence characteristics, evolutionary conservation, and physicochemical properties. Three of these candidates were subsequently synthesized and evaluated for antimicrobial activity against bacterial pathogens, and all three exhibited inhibitory activity against at least one tested bacterium. These findings expand the set of candidate AMP sequences identified in *R. amurensis* and provide insights into skin defense in this species. The identified AMP candidates provide a resource for future studies of amphibian host defense and natural AMP discovery.

## Figures and Tables

**Table 1:** Summary of RNA-Seq sequencing data.

Sample	ReadSum	BaseSum	GC (%)	N (%)	Q20 (%)	Cycle Q20 (%)	Q30 (%)
ZA_1	23,714,708	7,114,412,400	44.63	0.00	97.24	100	92.84
ZA_3	23,169,874	6,950,962,200	44.86	0.00	97.33	100	93.15
ZA_8	24,065,148	7,219,544,400	45.24	0.00	97.3	100	93.1
ZB_1	23,974,716	7,192,414,800	45.15	0.00	97.27	100	93.01
ZB_3	21,354,377	6,406,313,100	44.69	0.00	97.09	100	92.57
ZB_8	23,393,569	7,018,070,700	44.01	0.00	97.05	100	92.64

Sample: Sample ID; ReadSum: Total number of paired-end reads in clean data; BaseSum: Total number of bases in clean data; GC (%): GC content, the percentage of G and C bases in clean data; N (%): Percentage of N bases in clean data; Q20 (%): Percentage of bases with a quality score ≥ 20; Q30 (%): Percentage of bases with a quality score ≥ 30.

**Table 2:** Result of annotation.

Anno_Database	Annotated_Number	300 <= Length < 1000 bp	Length >= 1000 bp
COG_Annotation	7940	2731	5209
GO_Annotation	15,884	5421	10,463
KEGG_Annotation	28,973	13,564	15,409
KOG_Annotation	15,867	5527	10,340
Pfam_Annotation	18,312	5618	12,694
Swissprot_Annotation	16,987	5872	11,115
nr_Annotation	41,664	23,695	17,969
All_Annotated	42,555	24,437	18,118

Annotated databases: Functional databases used for annotation; Unigene: Total number of Unigenes annotated to the corresponding database; 300 ≤ Length < 1000 bp: Number of Unigenes annotated to the database with lengths ranging from 300 bp to 1000 bp; Length ≥ 1000 bp: Number of Unigenes annotated to the database with lengths ≥ 1000 bp.

**Table 3:** Annotation and differential expression information of AMP-encoding transcripts identified among DEGs.

Gene ID	AMP Classification	PFAM Description	BP Description	Regulation	log2FC (ZBvsZA)	q-Value (ZBvsZA)
Cluster-18722.2461	Beta defensin	Beta defensin	defense response	UP	2.6856	0.064853
Cluster-18722.28442	Ocellatin/Frog antimicrobial peptide	Ocellatin family/Frog antimicrobial peptide	defense response	UP	1.4259	0.055885
Cluster-18722.31071	Brevenin/Frog antimicrobial peptide	Frog antimicrobial peptide	defense response to other organism	UP	1.0127	0.99959
Cluster-18722.43229	Amurin-3 precursor	Frog antimicrobial peptide	defense response to other organisms	DOWN	−6.7371	0.05178
Cluster-18722.27667	Amurin-6AM	Frog antimicrobial peptide	defense response	UP	1.2108	0.99959
Cluster-18722.185	Beta defensin 136	Beta defensin 136	defense response to bacterium	UP	2.006	0.99959

**Table 4:** Identification of AMP-encoding cDNA transcripts using CAMPR3, DRAMP, and cysteine motif scanning.

Method/Database	Number of AMP-Encoding Transcripts Predicted	Non-Redundant AMP-Encoding cDNA Transcripts
CAMPR3	45	79
DRAMP	67	
Cysteine motifs	68	

**Table 5:** High-confidence AMP candidates validated by CS-AMPPred and primer-based screening.

Method/Database	Number of AMP-Encoding Transcripts Predicted	Total Number of Unique AMP-Encoding Transcripts
CS-AMPPred	79	151
Primer blast	72	

**Table 6:** Statistics of mature antimicrobial peptides identified by model-based scanning and primer-based alignment.

Method/Database	Number of AMP-Encoding cDNA Transcripts	Number of Mature AMPs	Total Number of Mature AMPs
Model-based scanning	79	74	90
Primer-based alignment	72	16	

**Table 7:** Mature sequences and basic physicochemical properties.

Peptide ID	Mature Amino Acid Sequence	Amino Acid Count	pI	MW (Da)	Net Charge
T2	HEELNHTPIFACDPLRAVQNILPKAICKKIKQSHSTLYVKFVTR	44	9.51	5089.01	5
T62	GKEPGCRTRFCWCALSWMPWSASQMRLERMRSCSPQHFRSS	41	9.88	4878.66	5
T17	AEDIIKPGKCPVRTEPCKVVLRPMCKRDGDCLQDLKCCYSCGKKCLSPY	49	8.69	5492.60	4
T26	SNNCANVCMQDKFEPTSVGKKKIHGFVVRMSDRL	34	9.30	3840.47	3
T74	CHVTCHMLRIVTCHVTCHMLRIVTCHVTCHMLRIVTWHVTCHML	44	8.63	5210.44	3
T19	ADTSSVGPCISWAREGPGVMSAACCKLVLKGSR	33	8.69	3337.89	2

**Table 8:** Predicted stability and hydropathy parameters and PROB scores.

Peptide ID	Half-Life (M)	Half-Life (Y)	Half-Life (E)	Instability Index	Aliphatic Index	GRAVY	PROB Score	Hydrophobicity	Hydrophilicity
T2	3.5	0.17	10	61.88	97.5	−0.241	0.47	0.86	−0.17
T62	30	20	10	81.94	23.9	−0.737	0.46	1.11	−0.29
T17	4.4	20	10	64.75	67.55	−0.39	0.37	0.96	−0.25
T26	1.9	20	10	28.86	60.00	−0.485	0.44	0.69	−0.24
T74	1.2	20	10	57.44	108.18	0.87	0.48	0.59	0
T19	4.4	20	10	23.74	73.94	0.215	0.49	0.62	−0.09

Half-life (M/Y/E) denotes the half-life in mammalian, yeast, and *E. coli* systems, respectively. GRAVY, Grand Average of Hydropathy; PROB score, a standardized score obtained from support vector machine (SVM) prediction, ranging from 0 to 1. A score closer to 1 indicates a higher potential for hemolytic activity, while a score closer to 0 indicates a lower potential for hemolytic activity.

**Table 9:** Statistical results of antibacterial effects of 3 antimicrobial peptides.

AMP	*E. coli*	*Salmonella* sp.	*A. hydrophila*	*S. aureus*
T2	+	+	+	-
T19	+	+	+	+
T26	+	+	+	-

+ indicates a clear inhibition zone; - indicates an unclear or absent inhibition zone.

**Table 10:** MIC values of T2 against four bacterial strains.

Cathelicidin-DM (μg/mL)	4	8	16	32	64	128	256	512
*E. coli*	-	-	-	-	-	-	-	+
*Salmonella* sp.	-	-	-	-	-	+	+	+
*A. hydrophila*	-	-	-	-	+	+	+	+
*S. aureus*	-	-	-	-	-	-	-	-

“+” indicates that the bacterial suspension was visibly clear, “-” indicates that the bacterial suspension was visibly turbid.

**Table 11:** MIC values of T19 against four bacterial strains.

Cathelicidin-DM (μg/mL)	4	8	16	32	64	128	256	512
*E. coli*	-	-	-	-	+	+	+	+
*Salmonella* sp.	-	-	-	-	-	+	+	+
*A. hydrophila*	-	-	-	-	-	+	+	+
*S. aureus*	-	-	-	-	-	+	+	+

“+” indicates that the bacterial suspension was visibly clear, “-” indicates that the bacterial suspension was visibly turbid.

**Table 12:** MIC values of T26 against four bacterial strains.

Cathelicidin-DM (μg/mL)	4	8	16	32	64	128	256	512
*E. coli*	-	-	-	-	+	+	+	+
*Salmonella* sp.	-	-	-	-	+	+	+	+
*A. hydrophila*	-	-	-	-	-	+	+	+
*S. aureus*	-	-	-	-	-	-	-	-

“+” indicates that the bacterial suspension was visibly clear, “-” indicates that the bacterial suspension was visibly turbid.

## Data Availability

The data presented in this study are available upon request from the corresponding authors.

## Supplementary Materials

The following supporting information can be downloaded at: https://www.mdpi.com/article/10.3390/biology15171479/s1, Figure S1: Phylogenetic tree of candidate antimicrobial peptides from *Rana amurensis*; Figure S2: AlphaFold2-predicted three-dimensional structures of three validated antimicrobial peptides identified from *Rana amurensis* skin transcriptome; Figure S3: Antimicrobial activity of AMPs from *Rana amurensis* skin. (a–d) Representative images of inhibition zones for three AMPs against *E. coli* (a), *Salmonella* sp. (b), *A. hydrophila* (c), and *S. aureus* (d). Red arrows indicate T2, yellow arrows indicate T26, blue arrows indicate T19, and green arrows indicate the control group. Table S1: Representative upregulated immune-related genes among differentially expressed genes; Table S2: Differentially expressed genes associated with the Toll-like receptor signaling pathway.
