# Supplementary material for: Preliminary Identification and Characterization of Antimicrobial Peptides from the Skin of Rana amurensis Based on Transcriptome Sequencing
Source: Biology (Basel). 2026 Sep 1;15(17):1479. doi: 10.3390/biology15171479 (PMC13565280; doi:10.3390/biology15171479)
Supplement: Supplementary file 1 [file biology-15-01479-s001.zip › Supplementary Figure S1.pdf]

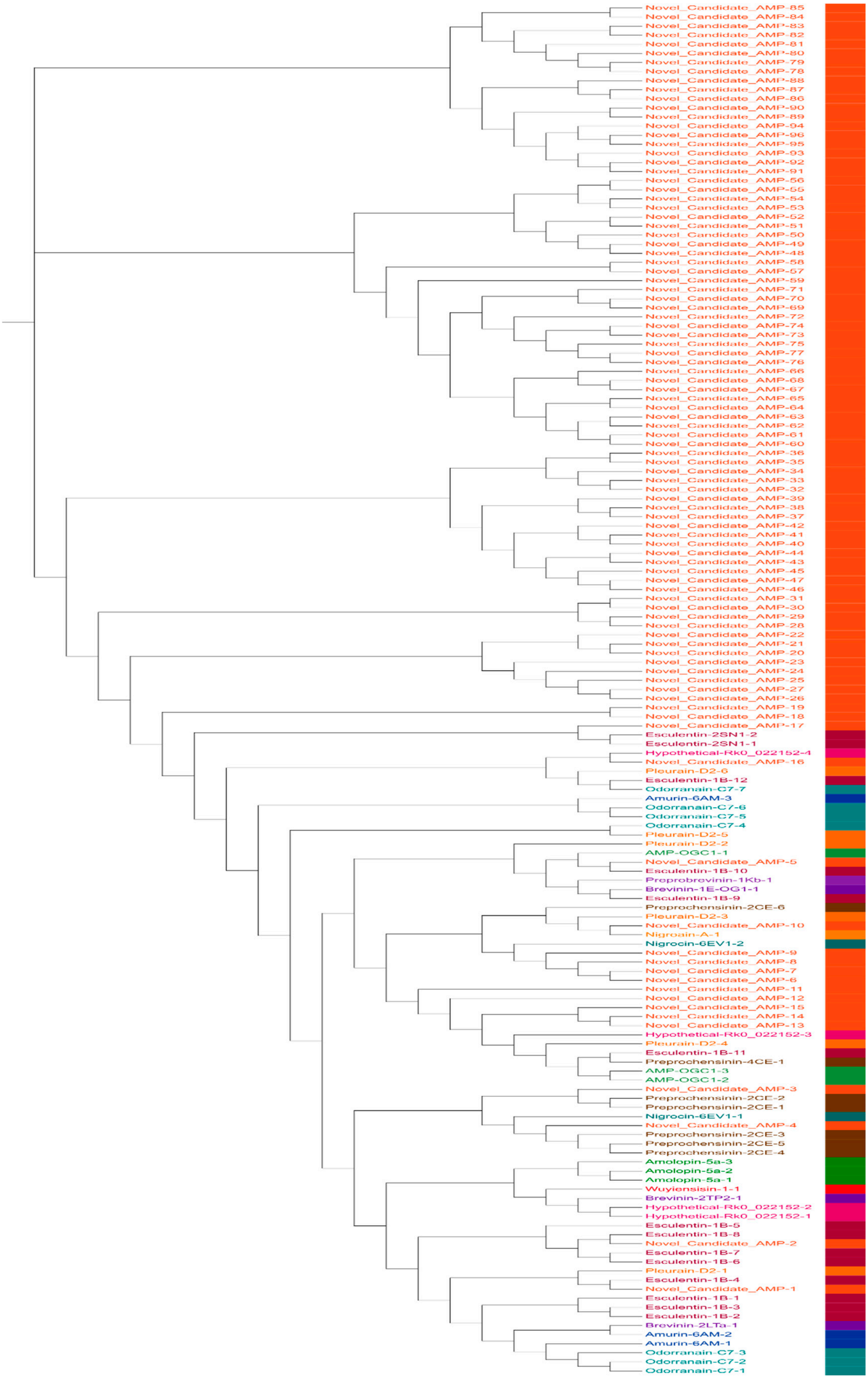

Figure S1. Phylogenetic tree of candidate antimicrobial peptides from *Rana amurensis*.
