# Supplementary material for: Preliminary Identification and Characterization of Antimicrobial Peptides from the Skin of Rana amurensis Based on Transcriptome Sequencing
Source: Biology (Basel). 2026 Sep 1;15(17):1479. doi: 10.3390/biology15171479 (PMC13565280; doi:10.3390/biology15171479)
Supplement: Supplementary file 1 [file biology-15-01479-s001.zip › Supplementary Figure S3.pdf]

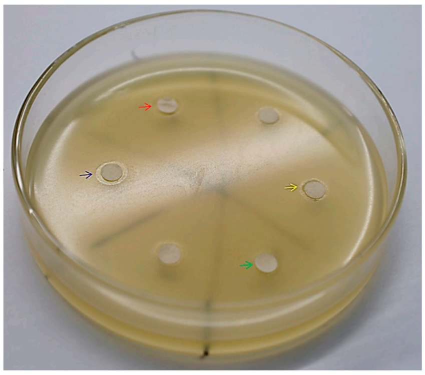

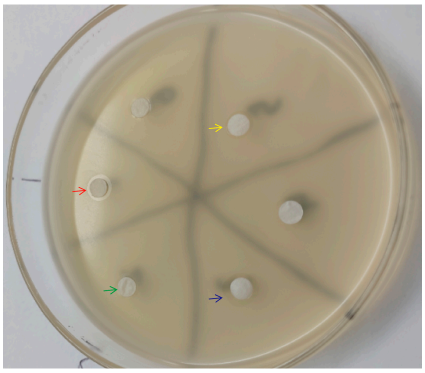

(**a**) (**b**)

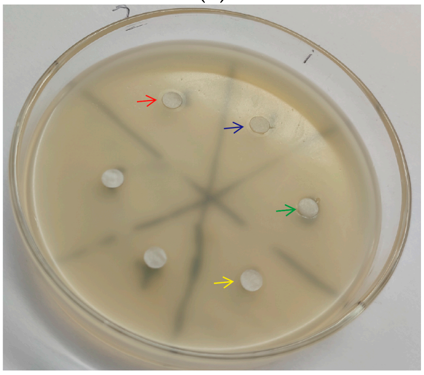

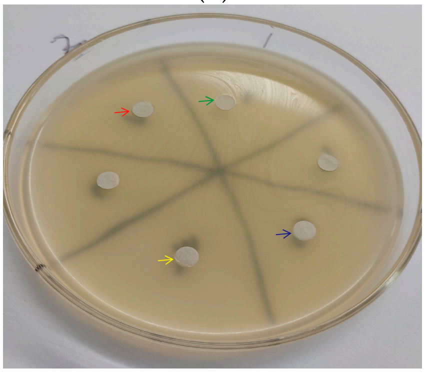

(**c**) (**d**)

Figure S3. Antimicrobial activity of AMPs from *Rana amurensis* skin. (a–d) Representative images of inhibition zones for three AMPs against *E. coli* (a), *Salmonella sp* (b), *A. hydrophila* (c), and *S. aureus* (d). Red arrows indicate T2, yellow arrows indicate T26, blue arrows indicate T19, and green arrows indicate the control group.
