# Supplementary material for: Preliminary Identification and Characterization of Antimicrobial Peptides from the Skin of Rana amurensis Based on Transcriptome Sequencing
Source: Biology (Basel). 2026 Sep 1;15(17):1479. doi: 10.3390/biology15171479 (PMC13565280; doi:10.3390/biology15171479)
Supplement: Supplementary file 1 [file biology-15-01479-s001.zip › Supplementary Table S1.pdf]

Table S1. Representative upregulated immune-related genes among differentially expressed genes.

Gene ID	Annotation	log2FC	q-value	Regulation	Category
Cluster-18722.15913	Interleukin-32	1.90	0.0266	UP	Cytokine
Cluster-18722.2461	Beta defensin	2.69	0.0649	UP	AMP
Cluster-18722.28442	Ocellatin family AMP	1.43	0.0559	UP	AMP
Cluster-18722.44452	LEAP-2 precursor	2.54	0.9996	UP	AMP
Cluster-18722.185	Beta defensin 136	2.01	0.9996	UP	AMP
Cluster-18722.3009	TLR13	1.08	0.9996	UP	Pattern recognition receptor
Cluster-10501.0	Complement C7	4.57	0.9996	UP	Complement
