# Supplementary material for: Preliminary Identification and Characterization of Antimicrobial Peptides from the Skin of Rana amurensis Based on Transcriptome Sequencing
Source: Biology (Basel). 2026 Sep 1;15(17):1479. doi: 10.3390/biology15171479 (PMC13565280; doi:10.3390/biology15171479)
Supplement: Supplementary file 1 [file biology-15-01479-s001.zip › Supplementary Table S2.pdf]

Table S2. Differentially expressed genes associated with the Toll-like receptor signaling pathway.

Gene ID	Annotation	KEGG pathway	log2FC	Regulation
Cluster-18722.10148	MAP2K7 (MKK7)	Toll-like receptor signaling pathway	-1.48	DOWN
Cluster-18722.19426	JUN (AP-1)	Toll-like receptor signaling pathway	0.75	UP
Cluster-18722.3009	TLR13	Toll-like receptor signaling pathway	1.08	UP
